# High Resolution Assessment of Spatio-Temporal Changes in O_2_ Concentration in Root-Pathogen Interaction

**DOI:** 10.3389/fmicb.2018.01491

**Published:** 2018-07-05

**Authors:** Mirco Rodeghiero, Simonetta Rubol, Alberto Bellin, Elena Turco, Giulia Molinatto, Damiano Gianelle, Ilaria Pertot

**Affiliations:** ^1^Sustainable Agro-Ecosystems and Bioresources Department, Research and Innovation Centre, Fondazione Edmund Mach, San Michele all'Adige, Italy; ^2^Energy Resources Engineering, Stanford University, Stanford, CA, United States; ^3^Department of Civil, Environmental and Mechanical Engineering, University of Trento, Trento, Italy; ^4^Agriculture, Food and Environment Centre (C3A), University of Trento, San Michele all'Adige, Italy; ^5^Department of Agricultural, Forest and Food Sciences, University of Turin, Turin, Italy

**Keywords:** fusarium, tomato, soil-borne pathogen, root respiration, planar optodes, spatial moments

## Abstract

Fusarium wilt, caused by the fungus *Fusarium oxysporum* f. sp. *lycopersici (Fol)*, is one of the most destructive soil-borne diseases of tomatoes. Infection takes place on the roots and the process starts with contact between the fungus and the roots hairs. To date, no detailed studies are available on metabolic activity in the early stages of the *Fol* and tomato root interaction. Spatial and temporal patterns of oxygen consumption could provide new insights into the dynamics of early colonization. Here, we combined planar optodes and spatial analysis to assess how tomato roots influence the metabolic activity and growth patterns of *Fol*. The results shows that the fungal metabolism, measured as oxygen consumption, increases within a few hours after the inoculation. Statistical analysis revealed that the fungus tends to growth toward the root, whereas, when the root is not present, the single elements of the fungus move with a Brownian motion (random). The combination of planar optodes and spatial analysis is a powerful new tool for assessing temporal and spatial dynamics in the early stages of root-pathogen interaction.

## Introduction

*Fusarium* wilt, caused by the soil-borne fungus *Fusarium oxysporum* f.sp. *lycopersici* (Sacc.) W.C. Snyder & H.N. Hans (*Fol*), is one of the most devastating diseases of the tomato. It is indeed responsible for severe losses in the greenhouse, open field crops and hydroponic cultures. *Fusarium oxysporum* f.sp. *lycopersici* infects tomato roots, starting from a contact with the root hairs and ending with the colonization and necrosis of the root tissue and wilting of the plant (Lagopodi et al., 2002; Mandal et al., 2009). Root colonization by *Fol* is, therefore, a crucial aspect in a successful pathogenesis (Lagopodi et al., 2002). Because of its economic importance, *Fol* has received considerable attention from researchers, especially in terms of root colonization patterns by pathogenic and non-pathogenic strains (Bao and Lazarovits, 2001; Lagopodi et al., 2002; Olivain et al., 2006), with and without the presence of microbial biocontrol agents (Bolwerk et al., 2003, 2005).

Fungal growth has traditionally been studied under the microscope, e.g., by measuring spore germ tube elongation during spore germination, by monitoring radial growth on jellified growth media in Petri dishes or by determining variations in fungal biomass (Cole, 1994; Dhingra and Sinclair, 1995). Moreover, colorimetric and spectrometric methods and microtitre plate assays have also been utilized (Hadacek and Greger, 2000). Oxygen consumption in three fungal species was recently studied with an indirect fluorimetric method, that was found to be highly sensitive and reliable in quantifying fungal activity (Nell et al., 2006). However, these studies did not visualize and quantify the early-stage interaction between the *Fol* mycelium and the tomato roots, especially in terms of spatial and temporal oxygen consumption patterns (i.e., oxidative metabolic activity, Novodvorska et al., 2016; Veillet et al., 2017).

Many biogeochemical processes occur in the roots. As an example, the roots influence the surrounding soil environment by releasing a blend of exudate compounds, which act as signals in plant-pathogen interaction (both for defense and/or pathogen stimulation) and can stimulate, for example, the germination of *Fol* microconidia (Bais et al., 2006; Steinkellner et al., 2009; Baetz and Martinoia, 2014). Besides consuming O_2_ for respiratory activities, root O_2_ emissions have been observed in plants inhabiting freshwater biomes, such as wetlands or flood-prone environments (McNamara and Mitchell, 1990; Colmer, 2003; Xu et al., 2013; Rudolph-Mohr et al., 2017).

Traditionally, root metabolic activity has been quantified by changes in oxygen concentration using microsensors, which provide local pointwise information, but are invasive. Thus this approach does not make it possible to study biological samples over a large area, or during prolonged periods of time (Tschiersch et al., 2011). Recent advances in imaging methodologies including cameras, scanners, fluorescence, and radiation-based techniques have enabled non-destructive exploration of root growth, including root interaction with soil-borne pathogens (Downie et al., 2015). Fluorescence-based optical sensors, such as non-invasive planar optodes, allow real time measurements of physiological processes offering manifold advantages over other methods including: spatial coverage (from mm^2^ to cm^2^), micrometric level of resolution and an extended period of measurement (from a few seconds to several days) (Tschiersch et al., 2012). Optodes have been successfully employed to quantify biological O_2_ exchange in seagrasses and other aquatic plants (Jovanovic et al., 2015; Larsen et al., 2015; Han et al., 2016), in the roots of terrestrial living plants (Blossfeld et al., 2011; Tschiersch et al., 2011; Rudolph et al., 2012; Rudolph-Mohr et al., 2015), in photosynthetically active leaves (Tschiersch et al., 2011; Ulqodry et al., 2016) and in the sapwood of woody trees (Gansert et al., 2001), as well as to assess oxidative metabolism in soils (Rubol et al., 2016) and biofilm (Rubol et al., 2018). Despite these research efforts, no planar optode studies have assessed soil-borne microorganism activity, either considering the pathogen alone or the pathogen interacting with plant roots. To fill this gap in research and explore the early stages of root-fungus interaction, we combined the use of non-invasive planar optode technology and geostatistical spatial analysis. We investigated how the temporal and spatial dynamics of *Fol* growth (assumed to be proportional to O_2_ consumption) are altered by the presence of tomato roots, and, *vice-versa*, how root physiology is affected by fungus colonization. The interaction was further examined using spatial analysis, to describe the dynamics of the fungus colony during root colonization and to quantify the nature of this interaction.

## Materials and methods

### Experimental setup

Seeds of *Lycopersicum esculentum* Mill. var. Marmande were surface disinfected in a 70% ethanol (30 s) and 1% sodium hydrochloride solution (1 min) (both from Sigma Aldrich, USA), carefully rinsed twice in sterile distilled water, and placed in a Petri dish (Ø = 90 mm) on 1/2-strength Murashige and Skoog medium (Murashige and Skoog, 1962). The seeds were then kept in a climate chamber at 23°C, with a day:night cycle of 14:10 h until the plant was at least 90 mm long (25 mm shoot and 65 mm root; reached in approximately 3–5 days).

**Figure 1:**
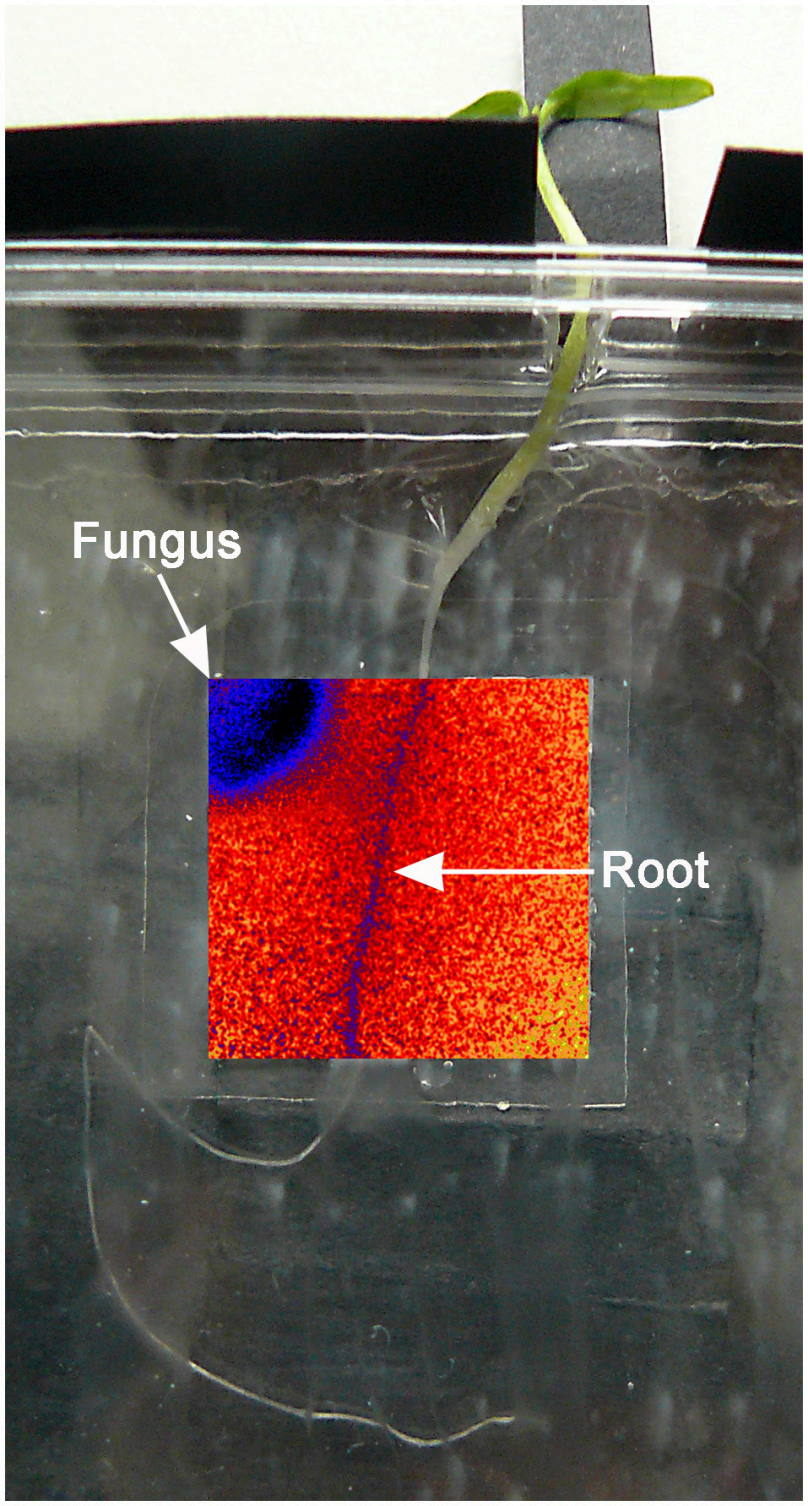
Testing system: the tomato plant was positioned with the root adherening to the optode and the aerial part protruding from the Petri dish through a small hole. The inserted overlay false color image shows the O_2_ distribution and position of the fungus.

The fungal material (*Fol* strain FGSC 9935) was acquired from Fungal Genetics Stock Center (US) and stored long-term at −80°C in glycerol (Sigma Aldrich). A conidial suspension obtained from the stock solution was transferred onto 1/4-strength potato dextrose agar (PDA; Oxoid, Italy), incubated for seven days at 25°C and the resulting colonies were used in the experiments. A square Petri dish (10 × 10 × 2 cm; L × W × H) was prepared the day before each measurement session, according to the following procedure. A 2 × 2 cm O_2_ sensor foil (SF-RPSu4 Oxygen sensor foil for imaging; Presens, 2013b) was glued to the inside of the Petri dish in the position where the root was supposed to grow and elongate during the experiment. The sensor foil was calibrated according to the Presens instruction manual (Presens, 2013a,b): a droplet of oxygen-free water obtained from a water solution of sodium sulfite (Na_2_SO_3_; Sigma Aldrich) and cobalt nitrate [Co(NO_3_)_2_; Sigma Aldrich] in nitric acid (HNO_3_; Sigma Aldrich) was used as the 0 point, whereas air saturated O_2_ (ambient air) was the reference for 100% O_2_. Then, a 0.3% agar (Sigma-Aldrich) solution, cooled to 55°C in order to avoid damages to the optode, was poured on the dish until it reached a thickness of 5 mm. Before pouring the medium into the Petri dish, the surface of the sensor foil was gently disinfected with 70% ethanol. Once the agar solution had cooled down to room temperature, a single tomato plant was carefully positioned inside the Petri dish, under the agar layer, so that the root adhered to the sensor foil whilst the stem and leaves were left to protrude outside, through a small hole made in the plastic (Figure 1). Similarly, at the same time, a 6 mm plug of fungal mycelium was placed under the agar layer in the upper right corner of the sensor foil (opposite to the aerial part of the plant). This root-fungus interaction experiment was repeated twice (on March 14 and May 21, 2014). Two controls were also performed: on the plant root without the fungus (on April 4, 2014) and on the fungus alone (on April 7, 2014), utilizing a 6 mm mycelium plug positioned in the center of the optode.

The Petri dish was sealed with Parafilm (Sigma Aldrich) along the perimeter and enclosed in a black cardboard to avoid light interference during measurement and at the same time, to protect the sensor foil from light. The shoot of the plant remained outside the black box to allow normal photosynthesis. A removable window the same size of the sensor foil was cut into the bottom side of the black box and covered with a removable cardboard flap, in order to allow the Vivisens detector unit to face the sensor foil. All the above mentioned operations were carried out in the dark and under sterile laminar flow to prevent any alteration of the sensor foil and/or biological contamination of the substrate.

### Image acquisition

The Vivisens detector unit (DU01 detector unit for spectral 2D read-out of fluorescent oxygen sensor foils; Presens, 2013b) was mounted on a microscope stand modified for the purpose: with this device the distance between the sample and the detector unit lenses was easily adjustable by turning a knob. A foam gasket was positioned between the detector unit and the Petri dish, in order to shield the system from interfering light sources (sunlight and or lab lamps). The distance between the microscope and the sample was set at about 5 cm, which was shown to be optimal in preliminary tests, to acquire images of the entire sensor foil. The detector unit was connected to a laptop, where VA1.12 VisiSens Analytical 1 software (Presens, 2013c) was programmed to measure and record images at 5 min intervals (See also Supplementary Video 1). During the experiment the air temperature in the laboratory was continuously recorded at 5 min intervals in close proximity to the Petri dish, with a temperature probe (thermocouple) connected to a CR23X data-logger (Campbell Scientific, USA).

### Images post-processing

Oxygen measurements were expressed as the percentage of O_2_ saturation in freshwater at atmospheric equilibrium (% air saturation). Oxygen images were first analyzed with image processing software (VisiSens Analytical 1 VA1.12-RC05; Presens, 2013c), which calculates the ratio of red to green in the emitted fluorescence response (the so-called *R*-value) provided by the color channels of the CMOS (complementary metal-oxide-semiconductor) chip (Presens, 2013c). The RGB images were calibrated by using the reference images obtained as described above and transformed into a 8-bit gray scale (256 levels of luminance). In the resulting figures white and black correspond to 0 and 100% O_2_ saturation, respectively. Subsequently, the images were cropped to remove the crown external to the sensor foil and transformed into a matrix of O_2_% air saturation using the Matlab function *imread* (MathWorks, 2012).

### Determination of O_2_ consumption rates

The O_2_ consumption rates were calculated based on the decline of O_2_% air saturation over time (i.e., the slope of the interpolating regression line) as reported by Tschiersch et al. (2011). We selected only specific time intervals where the regression line between O_2_% air saturation and time was highly significant (*p* < 0.01; *R*^2^ > 0.90). The slope (σ) was then used to calculate the O_2_ content in air-saturated water at temperature (θ) and pressure (*p*_*atm*_) according to the following equation (Presens, 2009):

(1) $$\begin{array}{l} c_{S}\;\left(p_{atm},\theta \right)=\frac{p_{atm}-p_{w}\left(\theta \right)}{p_{N}}\sigma 0.2095\alpha \left(\theta \right)\frac{M_{O2}}{V_{M}} \end{array}$$

where: *c*_*S*_ is the water O_2_ content in mg l^−1^; *p*_*w*_ is the water vapor pressure at the temperature θ; *p*_*N*_ is the standard pressure (1013.26 mbar); σ is the slope of the regression line; 0.2095 is the volume of O_2_ content in air; α *(*θ*)* is the Bunsen absorption coefficient (Benson and Krause, 1980) i.e., the volume of gas dissolved in a unit volume of solvent at standard partial pressure of the gas (1013.26 mbar) at the temperature θ; *M*_*O*2_ is the molecular mass of O_2_ and *V*_*M*_ is the molar volume of O_2_. The atmospheric pressure values (*p*_*atm*_) was recorded at the nearby weather station (San Michele all'Adige; 46.183498–11.120220) with a PTB220 digital barometer (Vaisala, Finland) whereas temperature (θ) was monitored close to the Petri dish (as explained above). Knowing the time step of image acquisition (5 min) and the surface area (selected for the root and fungus), we could then transform *c*_*S*_ from mg O_2_ l^−1^ into g O_2_ m^−2^ d^−1^. Since our agar medium was 0.3%, we performed the above calculations treating the medium as pure water: indeed, according to Van der Meeren et al. (2001), the diffusion coefficient of O_2_ in a 0.7–2.0% agar solution only decreases by 1.7 and 2.8%, respectively.

### Statistical analysis

**Figure 2:**
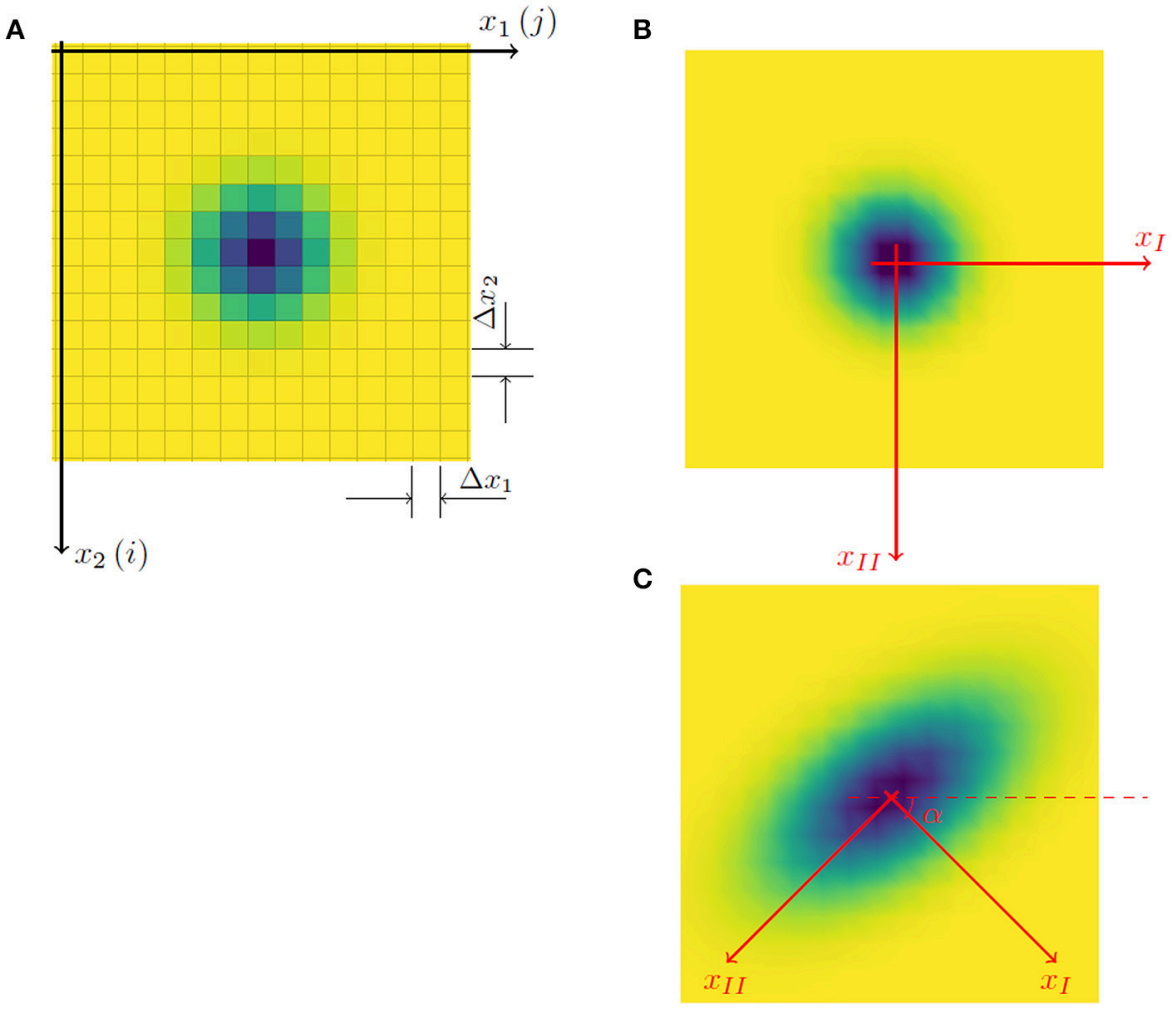
The reference system used to calculate the temporal dynamics of O_2_ concentration **(A)** was constituted by a computational grid with Δ_*x*1_, Δ_*x*2_ mesh coinciding with the pixel dimensions in the horizontal and vertical directions respectively (the color of the pixel represents the saturation deficit). **(B)** If the spatial growth of the fungus colony is uniform, the principal directions *x*_*I*_ and *x*_*II*_ coincide with *x*_1_ and *x*_2_ respectively, and angle α is 0 compared to the reference axis *x*_1_. **(C)** If the growth of the colony has a preferential direction (e.g., along axis *x*_*II*_) the principal directions are rotated clockwise by an angle α ≠ 0 (e.g., α = π/4). In all cases, the tensor of second moments is diagonal when evaluated in relation to axes *x*_*I*_ and *x*_*II*_ instead of *x*_1_ and *x*_2_, (i.e., *X*_*I,II*_ = 0 and *X*_*II,II*_ > *X*_*I,I*_).

**Figure 3:**
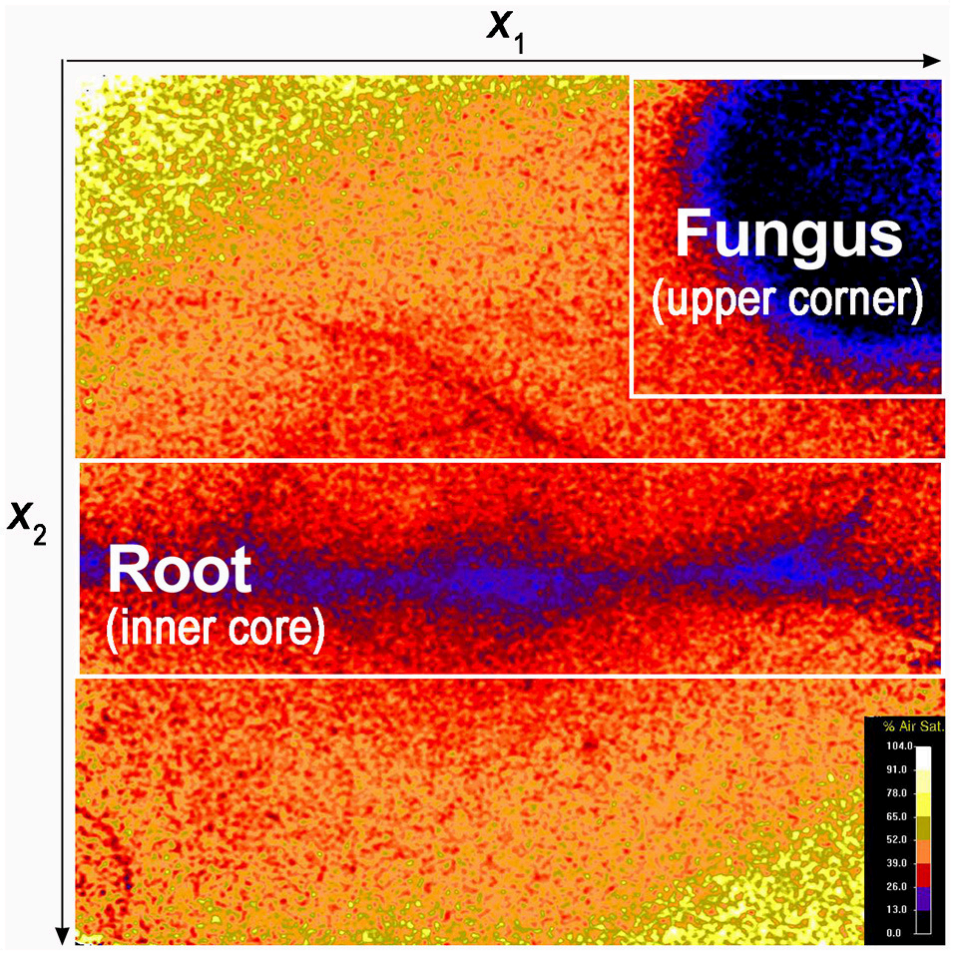
False color image showing O_2_ spatial distribution and the position of the root and fungus. The dynamics of O_2_ concentration were investigated in targeted portions of the sensor foil: i.e., the area surrounding the root (inner core) and the area surrounding the fungus (upper right corner).

The collected images were transformed into a matrix of oxygen concentration, with each value attributed to the center of the corresponding pixel. The position of each pixel is therefore identified by two indexes (i,j), referring respectively to the row and the column of the matrix (Figure 2) whereas *N*_1_ and *N*_2_ are the number of pixels along the horizontal and vertical directions, Δ_*x*1_ and Δ_*x*2_ are the respective dimensions of the pixels. The origin of the reference system was fixed in the upper left corner of the cropped image, with the *x*_1_ axis going from left to right and the *x*_2_ axis from top to bottom (Figure 3).

### Temporal dynamics of oxygen concentration

To quantify the dynamics of fungus and root O_2_ concentration, we selected targeted portions of the sensor foil: i.e., the area including the root and its adjacent surroundings (defined as the inner core) and the area including the fungus and its surroundings (upper corner or central part of the optode; Figure 3). We than assumed that the density of the fungus, was linearly proportional to the deficit in the O_2_ concentration:

(2) $$\begin{array}{l} \rho_{i,j}=\;\Delta C_{i,j}=\left(100-C_{i,j}\right) \end{array}$$

where ρ_*i,j*_ is the O_2_ deficit compared to saturation and *C*_*i,j*_ is the concentration of O_2_ expressed as a percentage of air saturation [the two indexes identify the position within the sensor foil, with *j*Δ*x*_1_ and *i*Δ*x*_2_ being the coordinates of the pixel center (i,j), having an O_2_ concentration *C*_*j,i*_. We also assumed that the mass of the fungus was proportional to its O_2_ consumption. Given the above assumptions, we characterized the fungus dynamics by computing the spatial moments of the oxygen deficit ρ.

### Spatial moments

Spatial moments were chosen because they are useful indicators for describing (i) in which direction the fungus is propagating, (ii) how the fungus grows (as expressed by second moments), and (iii) how the fungus is propagating (e.g., whether it is following a diffusive process or not). The first spatial moment illustrates the evolution in time of the center of mass of the fungus of coordinates ${\bar{x}}_{1}$ and ${\bar{x}}_{2}$, which is also described by the spatial distribution of the O_2_ deficit:

(3) $$\begin{array}{l} {\bar{x}}_{1}=\frac{1}{M}\sum_{i\;=\;1}^{N_{2}}\sum_{j\;=\;1}^{N_{1}}j\;\rho_{i,j}\Delta x_{1}\;\Delta x_{1}\;\Delta x_{2} \end{array}$$

(4) $$\begin{array}{l} {\bar{x}}_{2}=\frac{1}{M}\sum_{i\;=\;1}^{N_{2}}\sum_{j\;=\;1}^{N_{1}}i\;\rho_{i,j}\Delta x_{2}\;\Delta x_{1}\;\Delta x_{2} \end{array}$$

where $M=\overset{N_{2}}{\underset{i=1}{\sum }}\overset{N_{1}}{\underset{j=1}{\sum }}\rho_{i,j}\;\Delta x_{1}\;\Delta x_{2}$ is proportional to the total mass of the fungus. Similarly, the 2 s order moments *X*_11_, *X*_12_, and *X*_22_, which are descriptors of colony spatial distribution (i.e., spreading around the central mass) compared to the two reference axes *x*_1_ and *x*_2_, are given by:

(5) $$\begin{array}{l} X_{11}=\frac{1}{M}\sum_{i\;=\;1}^{N_{2}}\sum_{j\;=\;1}^{N_{1}}{\left(j\;\Delta x_{1}-{\bar{x}}_{1}\right)}^{2}\rho_{i,j} \end{array}$$

(6) $$\begin{array}{l} X_{22}=\frac{1}{M}\sum_{i\;=\;1}^{N_{2}}\sum_{j\;=\;1}^{N_{1}}{\left(i\;\Delta x_{2}-{\bar{x}}_{2}\right)}^{2}\rho_{i,j} \end{array}$$

(7) $$\begin{array}{l} X_{12}=\frac{1}{M}\sum_{i\;=\;1}^{N_{2}}\sum_{j\;=\;1}^{N_{1}}{\left(j\;\Delta x_{1}-{\bar{x}}_{1}\right)\left(i\;\Delta x_{2}-{\bar{x}}_{2}\right)\rho }_{i,j} \end{array}$$

*X*_11_, *X*_22_, *X*_12_, represent respectively longitudinal, transversal, and cross-directional spreading around the central mass of the fungus.

## Results

### O_2_ dynamics during root-fungus interaction

**Figure 4:**
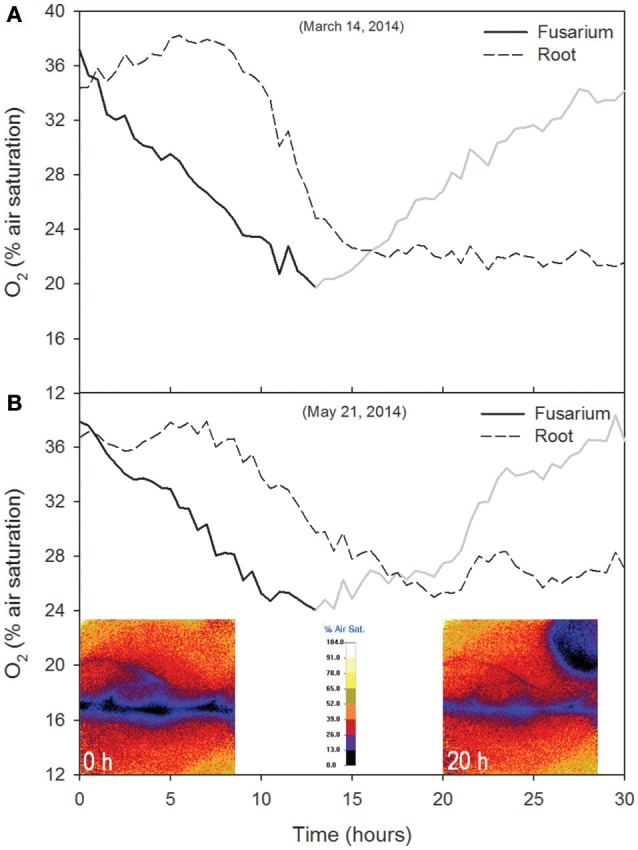
Time course of O_2_ (% air saturation) in the first **(A)** and second **(B)** root-fungus interaction tests. O_2_ data were averaged over 30 min and considered as a proxy for the oxidative metabolic activity of the fungus (*Fusarium oxysporum*) and the plant (*Licopersicum esculentum*). The gray part of the fusarium O_2_ line refers to the recovery phase. The false color images showing O_2_ spatial distribution and the position of the root and fungus over the optode are also reported **(B)**. These images were taken 0 and 20 h after the beginning of the experiment.

In the first experimental run, the O_2_% air saturation in the upper right corner of the sensor foil decreased for about 12 h, from 37.1 to 19.7% (Δ = 17.4%; Figure 4A). This was the consequence of an initially high fungal respiration rate (e.g., O_2_ consumption), which was quantified in 18.2 g O_2_ m^−2^ d^−1^, reaching a minimum and then recovering to the initial value (Figure 4A). The O_2_ level in the inner root core peaked at 38.2% at 5.5 h (i.e., *t* = 5.5 h) then decreased due to a high consumption rate, quantified as 19.9 g O_2_ m^−2^ d^−1^ (Figure 4A). Through visual inspection of the image sequence, we observed that the fungus colonized the root between 15 and 20 h from the beginning of the test; subsequently, changes in O_2_% air saturation were the result of the combined oxidative metabolic activity of two overlapping organisms, quantified as 0.5–1.2 g O_2_ m^−2^ d^−1^. When the root-fungus interaction experiment was repeated, similar behavior was observed (Figure 4B): fungal respiration increased, with O_2_ falling from 36.7% at the beginning of the experiment to 24.1% at *t* = 12 h (Δ = 12.6%) and recovered afterwards. The O_2_ concentration in the inner core (root) peaked at *t* = 8.5 h (37.9%) and then decreased (Figure 4B). The fungal and root respiration rates were 18.1 and 4.5 g O_2_ m^−2^ d^−1^, respectively.

**Figure 5:**
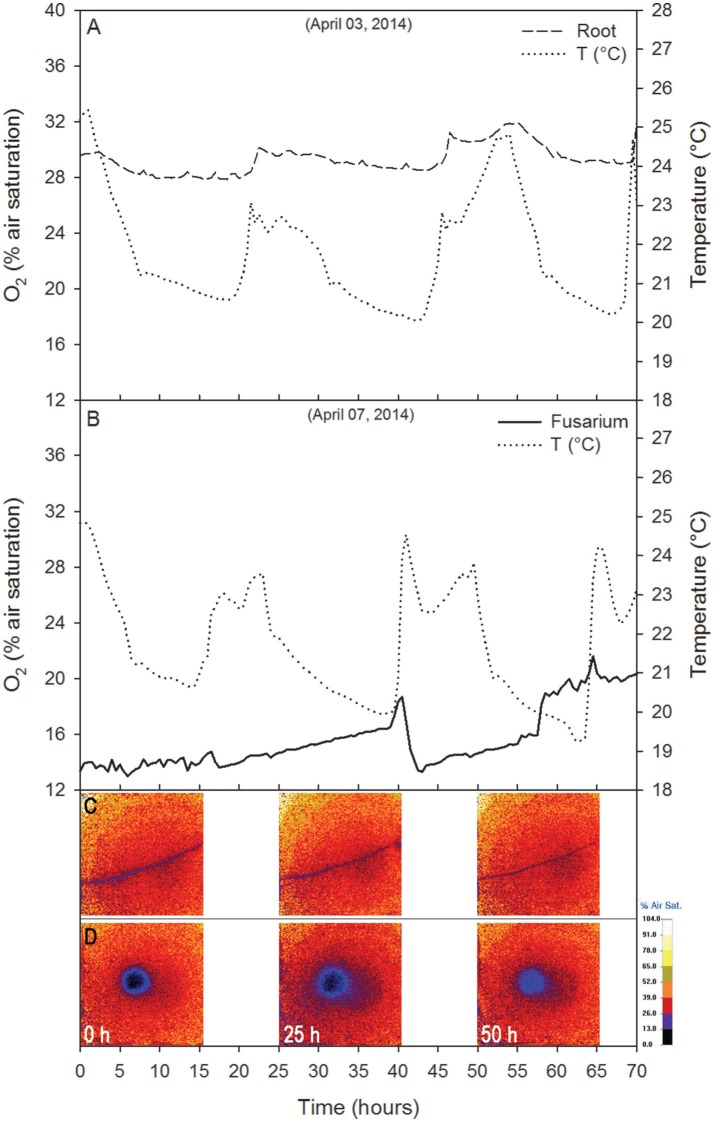
Control tests showing the time course of O_2_ (% air saturation) for the root alone **(A)** and the fungus alone **(B)**. The temperature recorded near the Petri dish is reported. Data are 30 min averages.

In the control with the plant alone, the root O_2_% air saturation varied between 27.9 and 32.2% (Δ = 4.3%; Figure 5A). The O_2_ peaks were in phase with temperature peaks (temperature ranged from 20 to 25.4°C; Δ = 5.4; correlation analysis: Pearson *r*^2^ = 0.44, *p* < 0.01, *N* = 141; from *t* = 0 to *t* = 70 h), whereas the concentration fluctuated breafly around 13.5% O_2_ for about 15 h (Figure 5B). A release phase followed, coinciding with a decrease in temperature. From *t* = 16 h to *t* = 70, the oxygen trend largely reflected the temperature trend (i.e., O_2_ concentration decreased with increasing temperature; correlation analysis: Pearson *r*^2^ = −0.05, *p* < 0.01, *N* = 141) with O_2_% air saturation ranging from 13.0 to 21.6% (Δ = 8.6%) and temperature ranging between 19.2 and 24.9°C (Δ = 5.7°C). Respiration rates varied between 37.4 and 101.0 g O_2_ m^−2^ d^−1^.

### Temporal and spatial activity of the fungus

**Figure 6:**
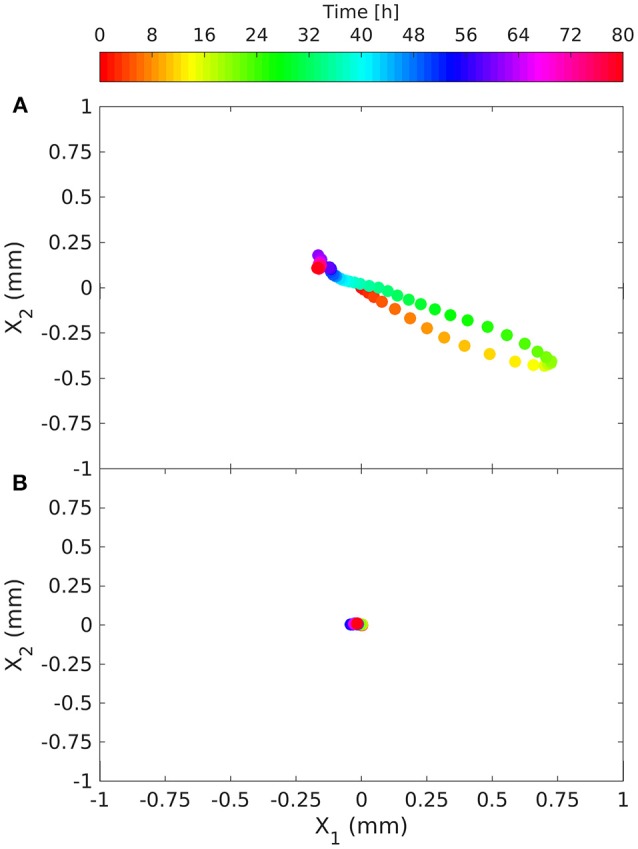
Time evolution of the barycenter (center of mass) of the fungus' O_2_ deficit relative to its initial position, in the presence of the root [**(A)**; March 14, 2014] and without the root **(B)**. The deficit is considered to be proportional to the mass of the fungus and provides information on fungal growth.

**Figure 7:**
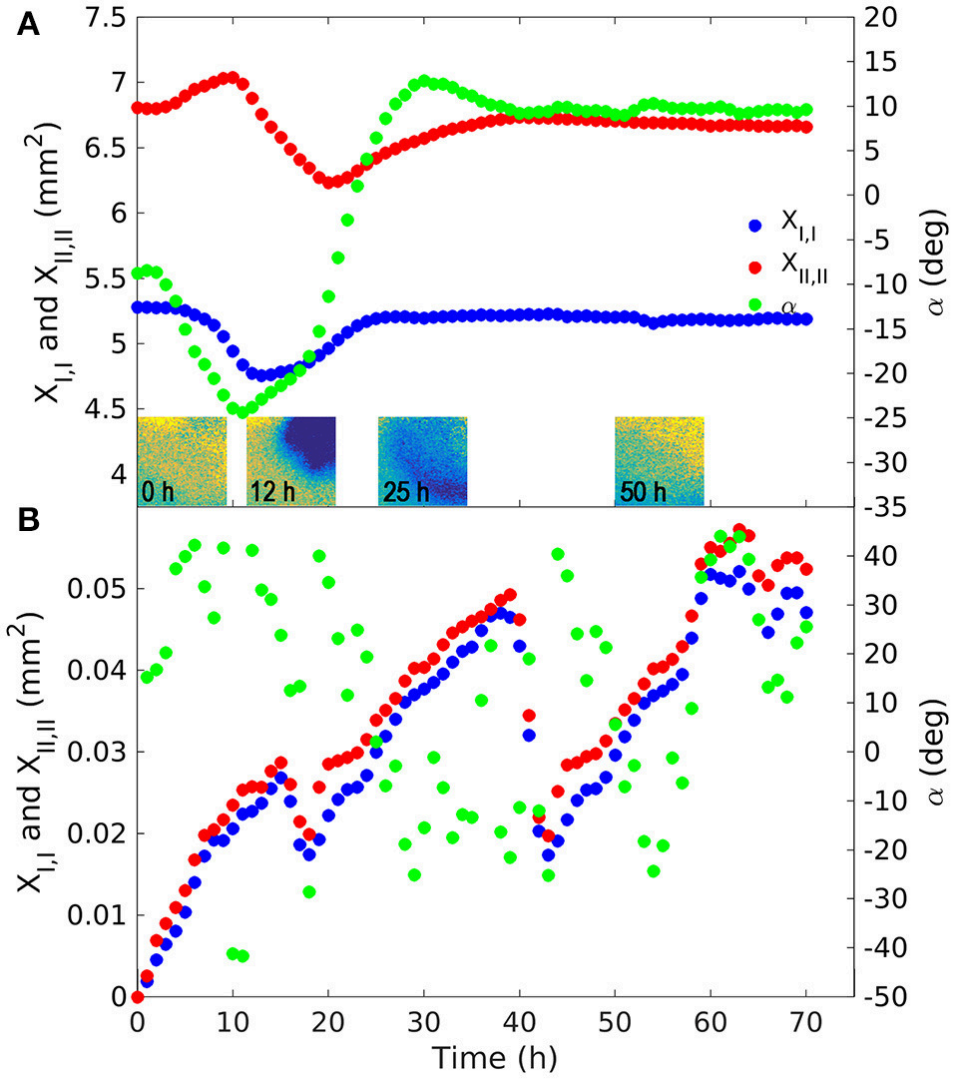
Principal second moments of the fungus body in the experiment with the root [**(A)**; March 14, 2014] and with the fungus alone **(B)**. The maximum principal second moment (*X*_*II,II*_) was computed with reference to axis *x*_*II*_, forming the α angle with the direction *x*_1_ of the reference system. The minimum principal moment (*X*_*I,I*_) was computed with reference to axis *x*_*I*_ orthogonal to *x*_*II*_, forming with it a local rotating orthogonal reference system. The tensor of moments of inertia is diagonal in relation to this reference system. These moments provide an indication of the dispersion around the centroid. The color figures in A are snapshots of the O_2_ deficit in the upper corner where the fungus was inoculated. Spatial density was assumed to be proportional to the oxygen deficit in comparison with saturation. The color of the pixels goes from yellow to blue representing low and high oxygen deficit (and fungus density) respectively. The secondary y axis indicates rotation angle α, expressed in degrees, of the principal axes compared to the *x*_1_ direction.

In the presence of the root, the trajectory of the fungus' center of mass rotated counter-clockwise with the maximum distance from the original position recorded 18 h after inoculation (Figure 6A). During this period the barycenter of the colony moved slightly away from the root (from left to right and upwards), the mass grew almost uniformly in the upper corner of the sensor foil. Initially the movement of the center of mass was very fast (after 12 h the distance traveled by the barycenter was about 0.8 mm), while it took about 60 h to return to its original position. At each time step the second moments were computed in the reference system (*x*_*I*_*, x*_*II*_) rotated by the angle α the reference system (*x*_1_*, x*_2_) (Figures 2B,C). Angle α was such as to make the tensor diagonal, with maximum second moment *X*_*II,II*_, oriented in direction *x*_*II*_ and minimum second moment *X*_*I,I*_ in direction *x*_*I*_. These two moments were obtained by making the matrix of second order moments diagonal. The second moment *X*_*II,II*_ first increased, reaching a peak 12 h after inoculation, then decreasing to a minimum at *t* = 22 h, and finally increasing again, reaching a stable value slightly lower than the initial one at *t* = 40 h. The second moment *X*_*I,I*_ was specular to *X*_*II,II*_ (increasing when *X*_*II,II*_ reduced and reducing when it increased) and reached a stable value after 25 h, well before the other moment (see Figure 7A). Angle α was first reduced by counterclockwise rotation, reaching a minimum after 12 h, when *X*_*II,II*_ was at its maximum, then increased with clockwise rotation to a maximum of about 15° at *t* = 30 h to finally stabilize at a value of 10° after 40 h by counterclockwise rotation.

In the control with the fungus alone, the dynamics were completely different. The center of mass was stationary (Figure 6B) and the second order moments grew linearly with time experiencing two sudden drops at 18 and 40 h (Figure 7B). These drops, in particular the second, indicated a contraction of the fungus metabolism caused by the decrease in temperature (see the Figure 5B). The linear increase in second order moments suggests that the fungus moved randomly with Brownian type movement leading to Fickian diffusion.

## Discussion

The comparable results obtained in the two root-fungus interaction experiments demonstrate the consistency and related reliability of the planar optode technique in monitoring O_2_ continuously, over a prolonged period of time and without perturbing the system. In both tests, an initially high level of fungal respiration (e.g., O_2_ consumption), peaking after 12 h was seen. The respiration rates were almost identical in the two control tests (18.2 and 18.1 g O_2_ m^−2^ d^−1^ respectively). A similar trend in *Fol* O_2_ consumption was reported by Nell et al. (2006). However, direct comparison with the values recorded by Nell et al. (2006) is not possible, since their data are expressed in terms of relative fluorescence units and the rates measured in our work are the result of different factors, such as root stimulation and presumably root exudates (Bais et al., 2006). Root exudates can indeed stimulate the germination of *Fol* microconidia (Steinkellner et al., 2005, 2009). The peak in fungal respiration was followed by a phase of lower respiration caused by a decrease in fungal metabolic activity present in the reference area (upper corner) due to the fact that the fungus moved out of the investigated area and colonized the root.

Root O_2_ consumption at the beginning of the first experiment (19.9 g O_2_ m^−2^ d^−1^) was similar to that of the fungus alone, whereas, in the second root-fungus experiment it was lower (4.5 g O_2_ m^−2^ d^−1^), probably due to lower physiological activity of the plant (Lai et al., 2016). The measured root respiration rates were of the same order of magnitude as those observed by Dong et al. (2011), in a wetland mesocosm for the aquatic plant *Acorus calamus* L. (14.4–30.3 g O_2_ m^−2^ d^−1^). The small number of available studies and the lack of experimental details (e.g., area, temperature, pressure) (Hadas and Okon, 1987; Tschiersch et al., 2012; Han et al., 2016; Rudolph-Mohr et al., 2017; Lenzewski et al., 2018) does not allow additional comparison with *in vitro* respiration rates. The change in O_2_% air saturation for the fungus alone (Δ = 8.6) was lower than that measured in the fungus during root-fungus interaction (12.6 < Δ < 17.4). This difference can be explained by the sudden peaks in air temperature recorded during the experiment with the fungus alone. The increase in temperature indeed promoted an increase in respiration rates (up to 101.0 g O_2_ m^−2^ d^−1^). The variation in O_2_% air saturation of the root alone was even lower (Δ = 4.3) than that measured during interaction. The low rates of O_2_ consumption can be related to potential root O_2_ emissions. Several studies have shown aerenchyma tissue development and O_2_ emissions from tomato roots and for other cultures as well, as a consequence of waterlogging (Kawase and Whitmoyer, 1980; McNamara and Mitchell, 1990; Xu et al., 2013; Vidoz et al., 2016).

Statistical analysis of the data allowed quantification of the aforementioned observations, making it possible to distinguish three distinct phases in particular: (i) a peak in oxygen consumption reached at 12 h occupying about 50% of the upper corner area; (ii) occupation of the whole area between 22 h (minimum of *X*_*II,II*_) and 25 h (maximum of *X*_*I,I*_); (iii) root colonization (Figure 7A). We cannot define the precise moment of contact between the fungus and root, because this would have required microscope investigation at a higher magnification, however we can say that this happened between 12 and 22 h (i.e., the fungus started to move after reaching peak activity). This behavior of *Fol* in colonizing tomato roots is in line with that observed by Lagopodi et al. (2002), although they used a destructive method, using a new biological sample for each subsequent observation and not showing the dynamics of metabolic activity for the two organisms. These complex dynamics ended at about 40 h, when both moments stabilized. From this time on the colony reduced its density uniformly in space. This behavior demonstrates that growth is stimulated by perception of the root and presumably by the diffusion of root exudates through the medium (Bécard and Piché, 1989).

In the experiment with the fungus alone, the barycenter of the object did not move, while second order moments along the principal directions grew linearly with time (Figures 6B, 7B respectively), apart from two sudden drops at 18 and 40 h due to abrupt changes in temperature (data not shown). Angle α oscillated between −40° and +40°. The linear, in time, growth of the second moments is indicative of a Brownian motion which is characterized by random movement of a population of particles (Einstein, 1956). This behavior was expected, since fungus has been shown to grow radially on jellified nutritional medium (Trinci, 1969).

## Conclusions

Overall, the planar optode technique proved to be a useful tool for investigating the O_2_ dynamics of root-fungus interaction, due to the flexibility of the system, which technically allows testing of the effect of virtually infinite perturbing factors in interaction and the possibility of studying other plant and fungal species. However, one limitation of the approach presented is the need to work in dark conditions to protect the sensor foil from light interference. This aspect, together with the need for a layer of water between the sample and the optode, make the approach unsuitable for open field studies at the moment. For the first time our work visualizes and quantifies the dynamics of O_2_ consumption and related metabolic activity characterizing root-fungus interaction, with the considerable advantage, as compared to standard methods, of not being destructive. Geostatistical analysis made it possible to describe and quantify the spatial motion of the fungus, which was stimulated by the presence of the root and oriented its growth. This situation was completely different in the experiment with the fungus alone, which was characterized by random Brownian motion.

We conclude that combining optodes and geostatistical analysis is a powerful tool for understanding the temporal and fine scale root-fungus interaction, especially to complement functional studies carried out with other approaches. Translation to real soils needs caution, given the significant effect of heterogeneity in soil and nutrient distribution, which affects the magnitude and direction of fungal growth.

## Author contributions

MR, SR, DG, and ET planned and designed the research. MR and ET performed experiments and conducted fieldwork. MR, SR, AB, and IP analyzed and interpreted the data. MR, SR, AB, ET, IP wrote the paper. MR, SR, AB, ET, GM, and IP revised the paper.

### Conflict of interest statement

The authors declare that the research was conducted in the absence of any commercial or financial relationships that could be construed as a potential conflict of interest.

## Supplementary material

Supplementary Video 1 False color image video showing the O2 spatial distribution during the interaction between 652 the root and the fungus from time *t* = 0 to *t* = 40 h. The O2 % air saturation decreases going 653 from light color (90–100% O2) to dark (0–13% O2).
